# Can mandatory monitoring in rental apartments effectively prevent legionellosis? A retrospective analysis of data from Regensburg with a review of the literature

**DOI:** 10.3205/dgkh000349

**Published:** 2020-06-30

**Authors:** Benedikt M. J. Lampl, Markus Lang, Saskia Wodnick

**Affiliations:** 1 Public Health Department, Regensburg, Germany

**Keywords:** Legionella pneumophila, mandatory monitoring, effectiveness, pneumonia, community acquired

## Abstract

**Background:** *Legionella pneumophila* can cause severe, often fatal, pneumonia in humans. Mandatory water sampling in commercially used buildings (mainly rental apartments) as regulated in the Drinking Water Ordinance (*Trinkwasserverordnung*) aim to protect the population against infection with Legionella. However, no data exist to date that could prove the effectiveness of the measures. At the same time, having the Public Health Department’s Infection Control Division deal with Legionalla is very time consuming.

**Methods:** A retrospective analysis of data from the city and district of Regensburg, a selective literature search, a prospective survey of workload using an anonymous questionnaire were performed.

**Results:** The evaluated data from the city/district of Regensburg suggest underreporting to a similar extent as shown by the RKI’s data in the compared period. Neither is the actual incidence known, nor can exposures be clearly determined in most cases. The exposure categories “travel” and “private/occupational” seem to be the most pertinent.

The potential public hazard of Legionella posed by domestic plumbing systems is unclear. A connection between exceeding the technical measurement limit (*Technischer Maßnahmenwert*, TMW) in routine tests in rental apartments and disease cases cannot be shown. A survey among non-medical personnel in the field of infection control and hygiene on the time spent on the topic of Legionella yielded a mean number of 39% of daily working hours for the observed 2-month period.

**Conclusion:** The data on incidence, exposure, and causality are incomplete. Evidence of effective protection by the current practice of sampling in apartment buildings could not be found. For many aspects, there are no unambiguous data in the literature. Restricting mandatory monitoring to certain public/commercial institutions should be discussed, given the high workload for the Public Health Department and the unproven protective effect. Further research on this topic is necessary.

## Zusammenfassung

**Hintergrund:** *Legionella pneumophila* kann beim Menschen eine schwere, oftmals tödlich verlaufende Pneumonie verursachen. Mit der in der Trinkwasserverordnung geregelten Untersuchungspflicht für gewerblich genutzte Objekte (v.a. Mietswohnungen) soll die Bevölkerung vor eine Infektion mit Legionellen geschützt werden. Bislang liegen jedoch keine Daten vor, die die Effektivität der Maßnahmen belegen. Gleichzeitig nimmt die Bearbeitung des Themenkomplexes Legionellen im Infektionsschutz des Öffentlichen Gesundheitsdienstes sehr viel Zeit in Anspruch.

**Methode:** Retrospektive Analyse von Daten aus der Stadt und dem Landkreis Regensburg, selektive Literaturrecherche und prospektive Erhebung zum Arbeitsaufwand mittels anonymen Fragebogens.

**Ergebnisse:** Die Daten aus Stadt/Landkreis Regensburg lassen eine ähnliche Untererfassung wie die Daten des RKI im Vergleichszeitraum vermuten. Weder ist die eigentliche Inzidenz bekannt, noch lassen sich in dem meisten Fällen eindeutige Expositionen ermitteln. Die Expositionskategorien „Reise“ und „privat/beruflich“ scheinen am bedeutsamsten zu sein.

Das Gefährdungspotential durch Legionellen in der Hausinstallation für die Bevölkerung ist unklar. Ein Zusammenhang von Überschreitungen des Technischen Maßnahmenwertes (TMW) in Routinebeprobungen in Mietswohnungen und Erkrankungsfällen lässt sich nicht herstellen. Eine Umfrage unter dem nichtärztlichen Personal im Sachgebiet Infektionsschutz und Hygiene zum Zeitaufwand für die Bearbeitung des Themas Legionellen erbrachte für den beobachteten Zeitraum von zwei Monaten einen mittleren Wert von 39% der täglichen Arbeitszeit.

**Schlussfolgerung:** Die Daten zu Inzidenz, Exposition und Kausalität sind lückenhaft. Hinweise auf eine effektive Schutzwirkung durch die aktuell geübte Praxis der Beprobung in Mietshäusern konnte nicht ermittelt werden. Zu vielen Aspekten liegen auch in der Literatur keine eindeutigen Daten vor. Eine Einschränkung der Untersuchungspflicht auf bestimmte öffentliche Bereiche sollte angesichts des hohen Arbeitsaufwandes seitens des Öffentlichen Gesundheitsdienstes und der nicht bewiesenen Schutzwirkung diskutiert werden. Eine weitere wissenschaftliche Beschäftigung mit der Thematik ist notwendig.

## Introduction

Legionella are gram-negative, aerobic bacteria with currently over 60 known species that occur globally in natural and anthropogenic aquatic habitats. They multiply intracellularly in amoebas or other protozoa; the temperature optimum for their growth is between 25°C and 45°C. All known legionella are classified as potentially pathogenic to humans. The most relevant species for humans in Europe is *Legionella pneumophila* with 16 serogroups, one of which causes approx. 90% of the diseases. Legionella can cause two distinct disease entities: legionellosis or Legionnaire’s disease and Pontiac fever. While the latter is a mild febrile illness with flu symptoms, legionellosis often is severe pneumonia with a fatality rate of 10% to 15% [1]. Inhalation of aerosols containing pathogens is considered to be the route of infection. Risk factors are immunosuppression, tobacco and alcohol abuse, age >40–50 years and male sex. Males in the age group mentioned are affected up to three times more often [2].

**Figure 1:**
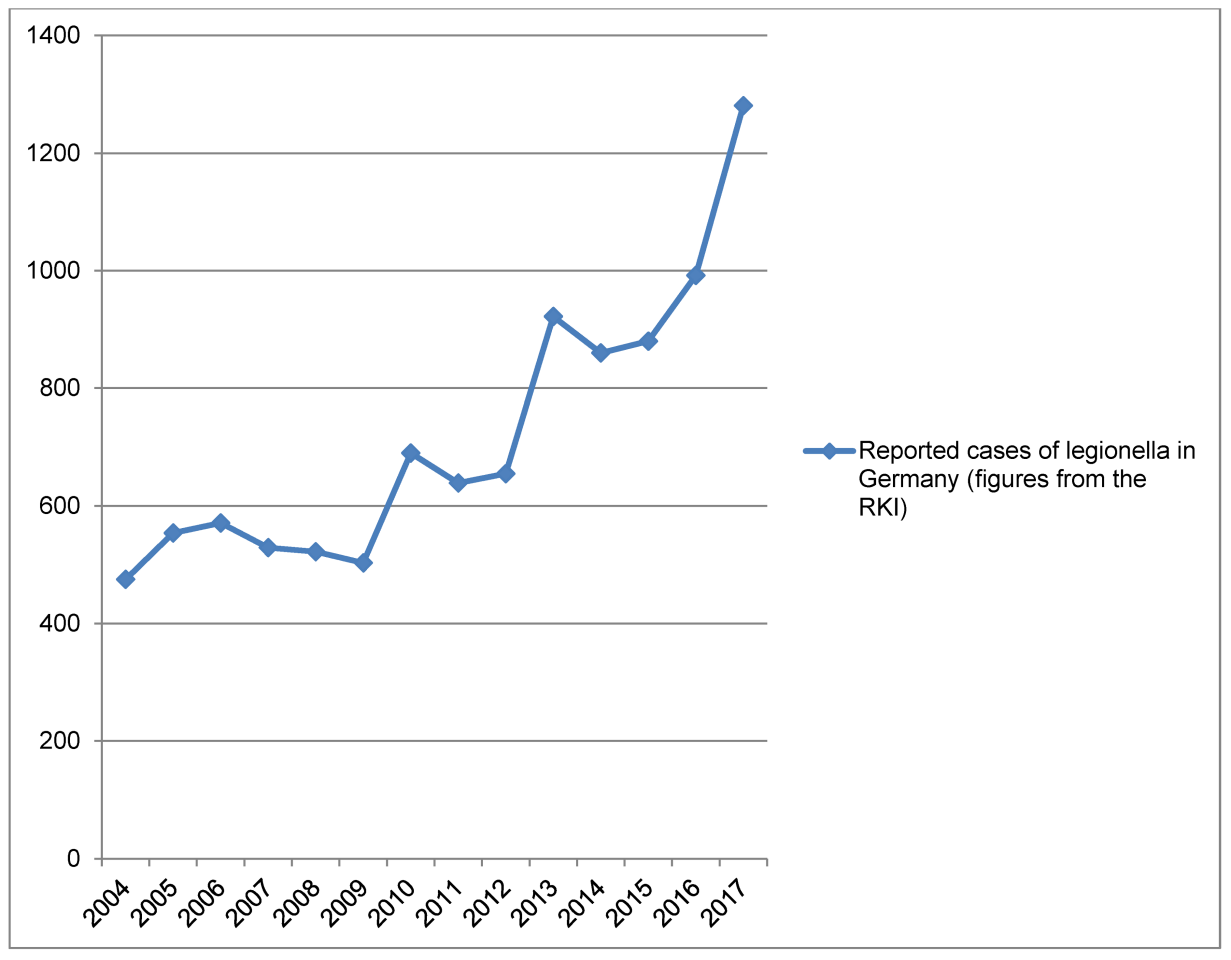
The figure shows the reported numbers of the RKI for legionellosis from 2004 to 2017 over time. From 2011 (coinciding with the change in mandatory testing according to the TrinkwV), a significant increase in the reported cases can be observed. The data were taken from the epidemiological yearbooks of infectious diseases 2004–2017.

In the 1980s, based on examination of a small number of pneumonia cases (community-acquired or nosocomial), legionella was estimated to be the cause of pneumonia in about 8% of cases [3], [4]. In 2008, Baum et al. examined the incidence of Legionella pneumonia in 2503 patients with community-acquired pneumonia (CAP) in the CAPNETZ study [5] and determined a percentage of 3.8%. Extrapolated, this means an annual number of around 15,000 to 30,000 cases of legionellosis [1], [4]. Based on the above-mentioned fatality rate, 1,500 to 4,500 deaths per year due to Legionella can be expected in Germany. This number is not reflected in the reported data in any way, which could be due to drastic underdiagnosis and/or underreporting on the one hand, or on the other hand to overestimation due to extrapolation. Data from the Robert Koch Institute (RKI) have shown that the number of reported cases has been increasing rapidly since 2011 (Figure 1 (Fig. 1), [6], [7], [8], [9], [10], [11], [12], [13], [14], [15], [16], [17], [18], [19]). It is completely unclear whether this increase in reported cases corresponds to an increased (or constant or even decreased) number of clinical cases.

Setting the technical measurement limit (*Technischer Maßnahmenwert*, TMW) to 100 cfu/100 ml and the hazard limit to 10,000 cfu/100 ml for legionella is controversial. It is not clear to what extent a specific hazard can be derived from a contamination found in the water system. The literature provided evidence that exposure to private home showering cannot be causally linked to legionella diseases on principle [20], [21]. The (historical) derivation of the TMW is understandable [2] to some extent but not empirically proven, and probably also not verifiable [22], [23], [24]. Thus, relevance of the TMW in terms of infection and hygiene remains unclear. The comparability of national studies is limited due to different drinking water regulations and technical requirements.

The original version of the Drinking Water Ordinance from 21/05/2001 initially specified an annual examination for Legionella in central heating systems of a plumbing system, from which water is provided to the public. With enaction of the 1^st^ Amendment of the Drinking Water Ordinance on 01/11 2011, this annual monitoring obligation was also extended to commercial operators, e.g., landlords of apartment buildings. The resulting additional effort for operators and the health authorities was justified by the deadly risk of legionella infections, especially in certain groups, such as elderly or immunosuppressed people, with reference to the findings of the CAPNETZ research project [5], [25]. 

The new obligation for non-public commercial large-scale hot water systems to report and act, associated with obligation to report the test results, led to capacity problems in the Public Health Departments. Thus, the Federal Ministry of Health enacted the 2^nd^ Amendment of the Drinking Water Ordinance (entry into force 14/12/2012) to relieve the public health authorities while maintaining the level of health protection. To this end, the investigation interval for commercial large-scale systems for heating drinking water was extended to three years and the former obligation to notify was eliminated. The limited capacities of the public health authorities should focus primarily on the protection of the public, including particularly vulnerable populations (e.g., in hospitals, old people’s homes, schools, and kindergartens) and secondarily on the private sector [26].

However, processing Legionella-related issues requires a great time investment by Public Health Departments’ Infection Control Divisions and binds human resources that are then not available for other important tasks. The main issue is the bureaucratic workload, due to mandatory testing in apartment buildings and the correspondence and telephone calls associated with it, which are to a large extent of administrative and legal nature.

## Objective

The aim of the present work was to evaluate the data collected within our jurisdiction in compliance with regulatory reporting of notifiable diseases and the Infection Protection Act, and to compare them with the data of the RKI for the same period of time. Taking the relevant literature into account, the question was examined whether it is possible to derive evidence that obligatory sampling, measurements and monitoring can effectively prevent legionellosis and associated deaths, especially in commercially used objects (i.e., rental apartments), given the high administrative work load and the considerable costs involved.

## Methods

The data collected on reported legionellosis cases for the city and district of Regensburg from the years 2004–2010 and 2011–2017 were evaluated retrospectively. Due to the incomplete datasets and the putative considerable underreporting of cases, age standardization was omitted, as was statistical testing. Data from the RKI were pooled for the above periods and used for comparison. A selective literature search was conducted in Medline using the terms “*Legionella pneumophila*”, “prevention”, “epidemiology”, “community acquired”, and relevant publications selected and used for discussion. Furthermore, the RKI recommendations were considered. In addition, a survey concerning the time spent on Legionella-related issues was carried out among the non-medical personnel in the field of infection control and hygiene (excluding tuberculosis care) in the Public Health Department of Regensburg. In the period 11/02/2019 to 05/04/2019, the daily working hours were recorded anonymously and an average was calculated from the respective percentage of the total working time of all respective employees.

## Results

**Figure 2:**
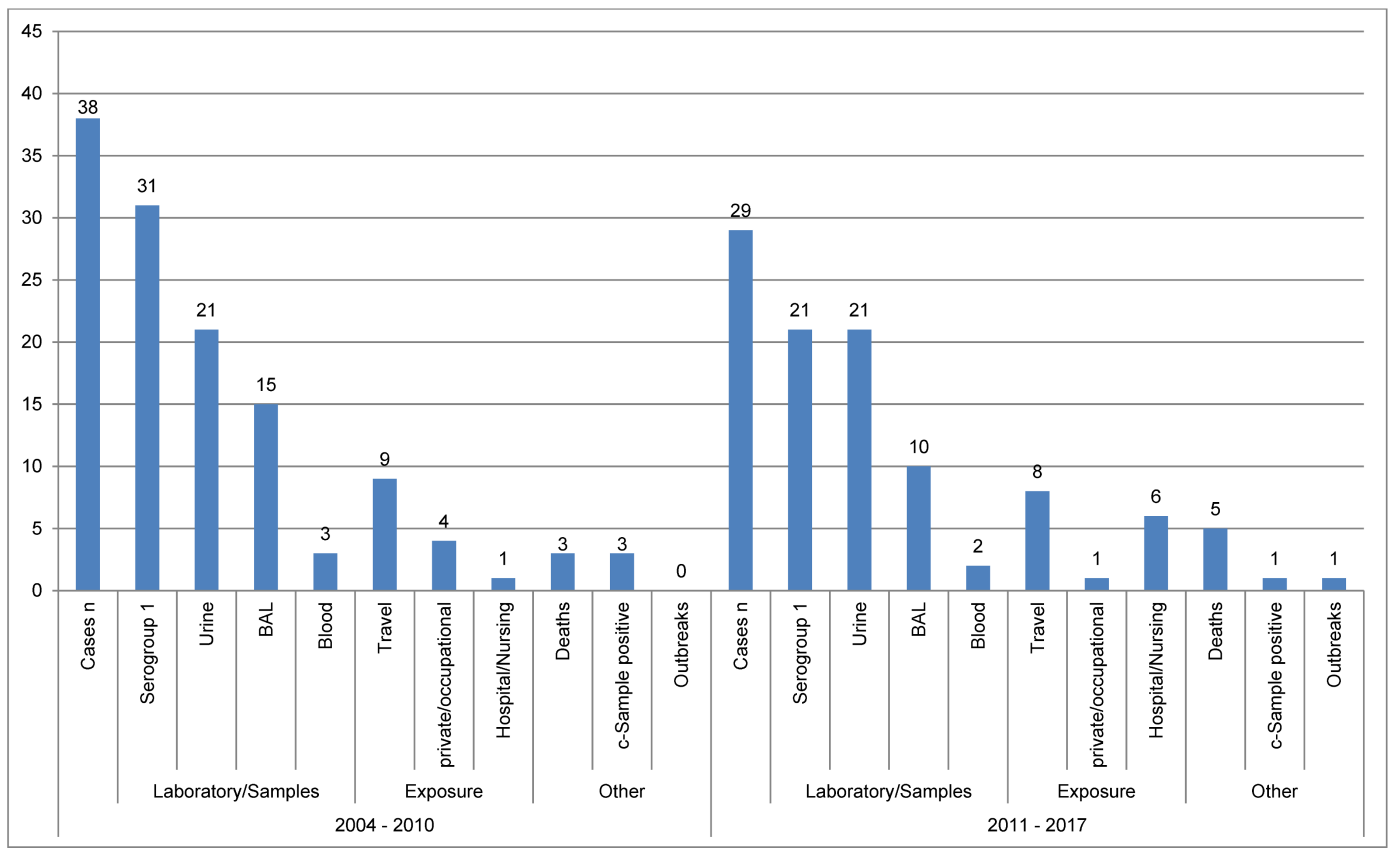
Data on legionellosis from the city/district of Regensburg 2004–2010 and 2011–2017

The reported legionellosis cases for the city/district of Regensburg in the periods 2004–2010 and 2011–2017 were considered. Figure 2 (Fig. 2) shows the data compared by time period. A total of 38 legionellosis cases were reported to the Regensburg Health Department from 2004 to 2010, and 29 cases from 2011 to 2017. In both periods, a relatively high proportion of travel-related cases (9 out of 38 [23.7%] and 8 out of 29 [27.6%]) was observed. This is the most common exposure in both reported periods. The fatality rate of the reported patients is 3 out of 38 (7.9%) for 2004–2011, and 5 out of 29 (17.2%) for the period 2011–2017. From 2011 to 2017, the proportion of “hospital/nursing” exposures (6 cases) was substantially higher than in the previous period. However, three of these cases can be assigned to an outbreak, which is shown separately.

From 2011 to 2017, only one occupational/private exposure could be determined. One of the samples taken according to “purpose c” (c-sample: sample of stagnatated water according to DIN EN ISO 19458, reflecting the status of the water at the tap as used by the consumer) was also positive during this period, whereas three positive c-samples were obtained in the comparison period. However, a proof of causality for the transmission was not conducted. Basically, the data are only fragmentary. The decline in the number of reported cases does not allow any statement about the true incidence or its development (increase/decrease in cases). Overall, similar numerical ratios can be seen in both periods, apart from the categories mentioned above. With regard to serotype prevalence, apart from serotype 1, no statements can be made, as other serotypes were recorded only sporadically. In the vast majority of cases, the detection of the infection consists in identifying the legionella antigen in urine (21 times), with which only serotype 1 can be detected [1] (total: 31 and 21 times, respectively; a positive urine test was regarded as detection of serogroup 1). 

**Figure 3:**
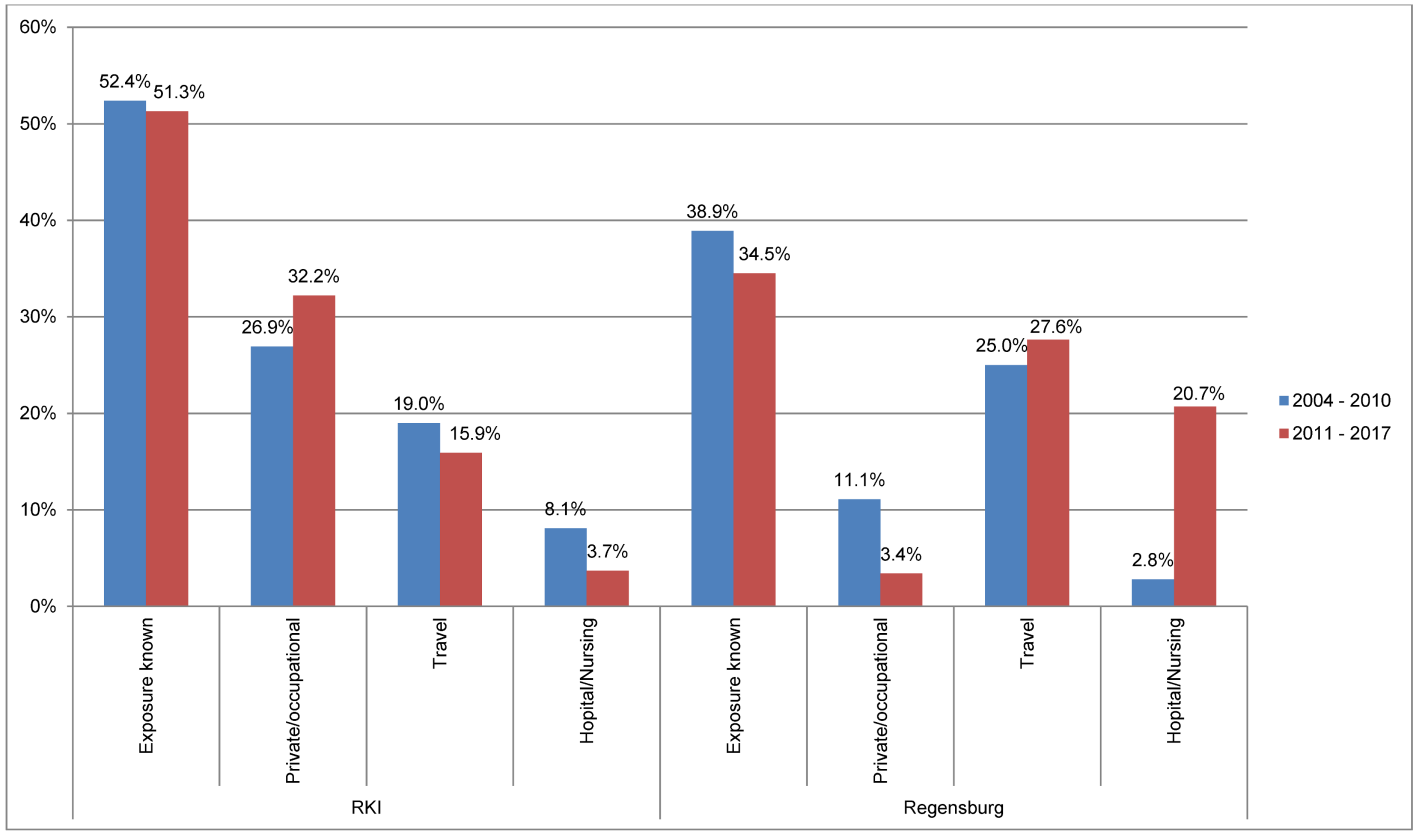
Data from the RKI and Regensburg on the determined exposure to Legionella pooled for the periods 2004 to 2010 and 2011 to 2017; percentages based on the total number of reported cases

The data from the Regensburg Health Department were also compared with data from the RKI. Figure 3 (Fig. 3) shows data from the RKI (cf. Table 1 (Tab. 1)) and the Health Department of Regensburg on the determined exposure to Legionella pooled for the periods 2004–2010 and 2011–2017. Percentages are shown in relation to all reported cases. The figures of the RKI show a similar distribution for the different exposure locations/situations for both periods. Exposure could only be determined for about 50% (RKI) and approximately 40% (Regensburg). It is striking that the RKI figures for “Hospital/Nursing” exposure dropped by more than half. In the city/district of Regensburg, on the other hand, “Hospital/Nursing” exposure was inversely related for both periods. The high proportion of 20.7% in the period from 2011 to 2017 can be partially explained by an outbreak with three patients (see Figure 2 (Fig. 2)). It is also noteworthy that the most frequent exposure in Regensburg is travel-related (25% and 27.6%), and that private/occupational exposure could only be determined once in 2011–2017 (3.4% of the cases). “Private/occupational” exposure could only be determined in 11.1% of the reported cases in Regensburg in 2004–2010; in the same period, this exposure was 26.9% in the RKI data. The proportion of the respective exposure type is approximately the same for the reported cases of the RKI and in Regensburg, with the exception of “private/occupational” exposure, which suggests that a similar percentage could also be expected for the latter exposure, even if this was not determinable. It should be noted that the two exposure types “private/occupational” (most frequent exposure according to RKI, 26.9% and 32.2%) and “travel” (most frequent exposure Regensburg 25% and 27.6%) are the most important. However, this information does not exactly equal defined temporal and spatial exposures, but only exposure categories. Statements about the actual source of infection are therefore not possible.

**Figure 4:**
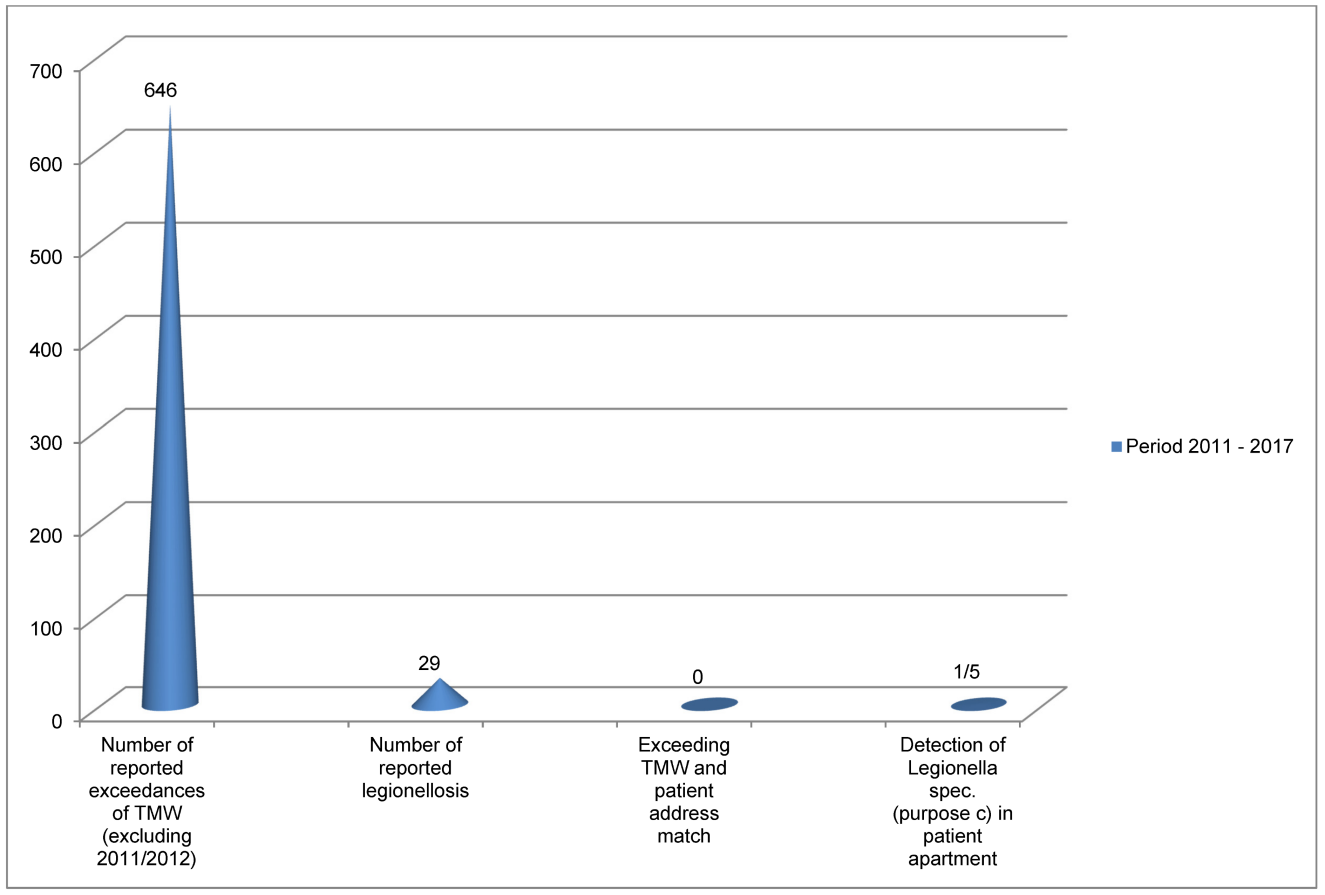
Data from the city/district of Regensburg on exceedance of the TMW and legionellosis from 2011–2017 (2011/12 no systematic recording)

With regard to the commercially used, i.e., rental objects, we investigated how often the TMW was reported to be exceeded. From 2011 to 2017, the total number of times the TMW was exceeded in 646 objects subject to mandatory testing (commercial/public) were reported to the Regensburg Health Department (Figure 4 (Fig. 4)). In the same period, the number of reported Legionellosis cases was 29. In none of the cases was it possible to match the residential address of the affected patient with an address from which a TMW exceedance was reported. In the one of the c-samples (5 cases), Legionella was detected: 200 cfu/100 ml were found in the sample from the shower hose/head. However, differentiation by GLISA rapid test revealed Legionella spp. The latex test was negative. Thus, *Legionella pneumophila* could not be detected. In the patient, the urine antigen test was positive (*Legionella pneumophila*, serotype 1); no further material was available.

The literature contains controversies and unanswered questions regarding important determinants of Legionella. For instance, virulence factors are not sufficiently characterized, there is a paradoxical dose-response relationship in connection with the internalization of Legionella by amoebae [1], and the role of aerosols formed during showering and the importance of exposure to this source in private homes are unclear [20], [21]. There are studies that deal experimentally with the formation and distribution of drops and aerosols formed during showering [27] as well as with mathematical exposure-risk models [28], [29], but due to the rather theoretical nature of the question, no pragmatic conclusions can be drawn from this. In Germany, unlike in other countries, air conditioning systems are of less importance as a reservoir in private households and thus as a source of infection. However, the role of humidifiers, inhalers, or flow heaters is unclear. There are no reliable data on the true incidence of legionellosis. Considerable knowledge gaps still exist with regard to the pathogen, the pathomechanism, hazard potential, and epidemiology [6], [7], [8], [9], [10], [11], [12], [13], [14], [15], [16], [17], [18], [19], [20], [21].

From 11/02/2019 to 05/04/2019, the working hours the hygiene staff (six hygiene controllers, one employee in infection protection) spent on legionella-related issues was recorded in the infection protection and hygiene unit (except tuberculosis care) (n=7). Every employee entered these working hours in a table at the end of each working day. Absolute numbers and percentages related to the daily working hours were determined and an average was formed from the percentages of each employee. Telephone calls, correspondence, discussions in the infection control/hygiene division and with the responsible public order and safety official, other legal and technical meetings, appointments etc. were taken into account. The average working time was 39% of the daily hours worked to process Legionella-related issues during the observation period.

## Discussion

Two fundamental questions arise in the debate: How dangerous are legionella bacteria for the majority of the population, also in view of the demographic development and the increasing number of immunosuppressed patients? And: Can legionella diseases be effectively prevented with the current legal situation and implementation, especially with the obligation for regular testing in rental apartments?

After evaluating the data from the city/district of Regensburg for the present work, it can be stated that the data are very incomplete and must be interpreted with caution. In this respect, they do not allow any reliable answers to the question asked at the start. However, evidence is available that does not necessarily support a protective effect on the population. In our data pool, addresses at which the TMW was reported to have been exceeded were in no case identical to the address of a patient suffering from legionellosis. This finding can mean:

1. No affected patient lived in an object subject to examination.
2. In the affected object, testing was not performed in spite of the law.
3. The affected object was examined but the TMW was not exceeded.
4. The affected object was examined, and the TMW was exceeded, but this was not reported.

Ultimately, however, it is not relevant why no exceedances of the TMW were reported; the fact is they were not reported. Thus, exceedances, if they existed, could not be registered, and the resulting measures, if they are effective at all, could not contribute to the prevention of legionellosis. On the other hand, the requirement that the competent authority must determine the obligation to examine each object would not be feasible, given the personnel/financial conditions. Consistent with this, the obligation to notify objects subject to mandatory monitoring was already abolished in 2012 (see above). The finding also means that residents from objects exceeding the TMW did not experience legionella disease (or were not reported). This may in turn mean that the technical measures (if they were performed) have prevented legionellosis or that exceedance of the TMW is irrelevant to the onset of the disease.

The survey on Legionella-related workload carried out among the hygiene staff in the field of infection protection and hygiene regarding shows – with all method-related restrictions (non-representative collective, relatively short observation period, interviewer bias, recall bias, consistency bias, desirability bias, etc.) – that the issue ties up a large part of working hours and human resources. This does not necessarily have to apply to other (state) health departments, especially in less urban areas. However, the figures for Regensburg make it clear that, given the shortage of personnel, resources may be shifted at the expense of other important tasks in the area of infection protection and hygiene, even assuming a method-related overestimation of personnel efforts. It must be kept in mind that Legionella is only a small part of the drinking water complex and the latter is only a small part of the range of infection protection and hygiene tasks.

As at the federal level, it must be assumed that underreporting of legionellosis (reporting bias) exists. This can be partially explained with the severe course of lethal legionellosis. Admission to the hospital with severe clinical symptoms sets a different diagnostic cascade in motion, whereas an outpatient presenting with milder symptoms may be given calculated antibiotic treatment without further diagnostics. Accordingly, this condition will be less likely to occur as a legionellosis laboratory report. This underregistration is generally difficult to manage, especially in the outpatient sector. Calculated antibiotic therapy is successful in some cases (Legionella are sensitive to gyrase inhibitors or macrolides [1], which are also used in the treatment of pneumonia of other etiology) even without attempting to detect pathogens or performing genotyping. In the hospital as well, merely urine testing for legionella antigen detection is often done. A proof of causality in the case of a positive result, e.g., in a c-sample from the domestic shower hose, cannot be provided, meaning that a positive Legionella test in the house plumbing system (of whatever concentration) and a positive antigen test in the urine do not allow the conclusion that the patient has been infected at home. This proof, if at all, can only be provided with sufficient certainty by an identical genotype in both samples. This, in turn, makes it difficult to predict the relevance of exposure in the home environment. The medical history of potential exposure and the attribution to one of the above-mentioned exposure categories must be considered questionable in many cases. This can be illustrated by an example: A patient suffering from legionellosis reports that s/he has been on vacation for two weeks in different places. Three days after returning home, s/he noticed symptoms. Now, what is the exposure: “travel”? Or “private/occupational” due to three weeks of unused shower at home (“improper use”)? Without genotyping material, the answer is only speculative, regardless of any species/antigen detection in a hotel/in the shower at home or in the affected patient. This fact also turned out to be problematic in the evaluation of our own data.

Almost no answers are available from what has been stated above, but there are a number of questions that require great research efforts to be answered. With regard to the postulated underassessment, the question arises as to whether we can even detect a decline in legionellosis diseases. It can be doubted whether a relaunch of the CAPNETZ study with its relatively small and therefore questionably representative number of cases could prove the effectiveness of the current measures in a longitudinal approach. Perhaps the LeTriWa study, which will be completed at the end of 2019, can shed light on some of the aspects mentioned [30].

For preventive and environmental medicine, the ALARA (“as low as reasonably achievable”) principle adopted from radiation protection applies. But what is *reasonably achievable* with regard to the concentration of Legionella and given the paradoxical dose-response relationship? Preventive health measures have high priority, as the course of legionella pneumonia can be dramatic [31], [32], [33]. Although the goal must be to prevent all these cases, the relevance of legionella prevention is questionable in relation to other preventable deaths. At the moment, it is speculative that we can effectively prevent disease/deaths from legionellosis by conducting regular laboratory testing in apartments. Thus, it should be discussed whether it would be appropriate to restrict the mandatory monitoring to public buildings, hospitals, retirement/nursing homes, cooling towers, hot tubs, etc. [34], as in neighboring countries (see Table 2 (Tab. 2), [35], [36], [37], [38], [39]), because the right to preventive protection for these buildings and facilities can be postulated as different from private apartments. Furthermore, the question arises whether there is a certain degree of individual responsibility for personal health with regard to the house plumbing system, especially since regular use and correct temperature regulation already considerably reduce risks in many cases. In this sense, would not alcohol and tobacco abstinence be suitable for preventing a large number of legionellosis as well as other pneumonia cases (together with better vaccination coverage for pneumococci in the respective risk group, [40])? In terms of preventive health protection, well-known public health strategies could be considered, such as information campaigns, brochures in general practitioners’ offices, etc. The objection can be raised that well-established protective measures are difficult to revoke; however, with good arguments it is possible. One argument would be that they have no proven benefit.

Finally, the question arises as to whether chlorination of drinking water should be considered if we assume a persistent health risk from Legionella in the drinking water (even if only in the domestic plumbing system).

Logically thinking through the reasoning behind the preventive concept of protection in terms of Legionella, it is not necessarily clear why one- and two-family houses *per se*, whose house plumbing system can in some cases be quite susceptible to Legionella colonization, are exempt from the obligatory monitoring. It cannot be assumed that older, male, diseased smokers or otherwise immunosuppressed patients always live in rental apartments, and young, healthy people with low risk always live in one- and two-family houses (with low pipeline volume). With a three-year examination interval, what can we say about any health risk and apart from the need for technical measures [41]? How safe can we feel with unremarkable findings from the systemic examination (once in three years), if a high level of contamination simultaneously exists in a shower hose that is never sampled?

## Conclusions

Exercising all caution regarding the many unanswered questions, there is to date no evidence that the current practice of legionella sampling in apartment buildings/private apartments and homes can effectively prevent legionella diseases or associated deaths. However, the time spent on the matter by the authorities and the costs are considerable. It is questionable whether proof of effectiveness can be provided at all. To be fair, it should be noted that there is likewise no reliable data that could prove the measures useless. From a scientific point of view, we should not be satisfied with the current situation. The present work only contains a retrospective data analysis and a small prospective, non-representative survey, and is therefore of limited significance due to its methodology, the underlying rudimentary data, and the individual evaluation of only a single Public Health Department. However, it can raise awareness of the problem and identify further research needs. Preventive health protection must prove the effectiveness of its measures and cannot rely on taking theoretically effective measures *optima fide*.

## Notes

### Competing interests

The authors declare that they have no competing interests.

## Figures and Tables

**Table 1:**
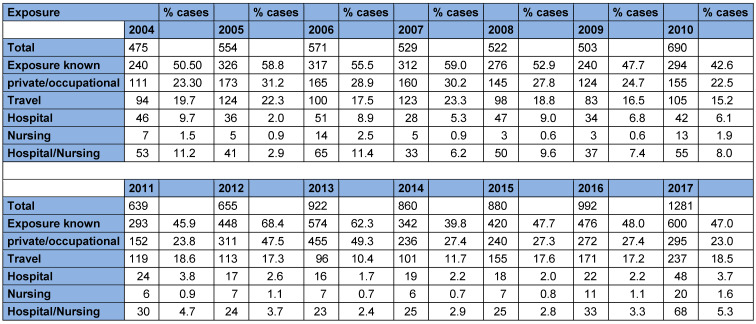
RKI figures on exposure to Legionella for the periods 2004–2010 and 2011–2017. The data were taken from the epidemiological yearbooks of infectious diseases 2004–2017. Percentages for the individual exposures were calculated on the total number of reported cases.

**Table 2:**
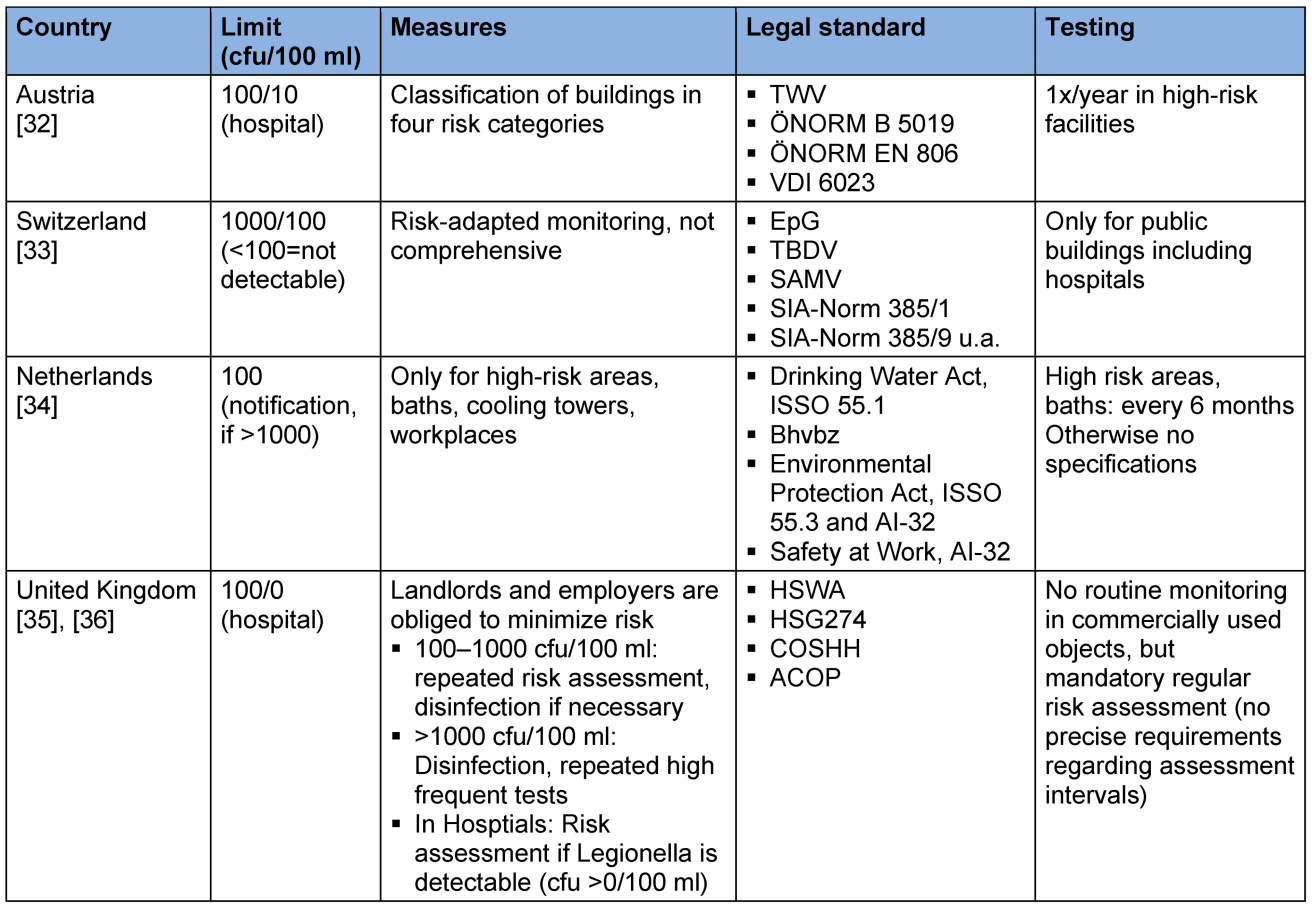
Limits, compulsory measures, legal standards and examination intervals in selected neighboring European countries
